# Protective effects of apocynin on damaged testes of rats exposed to methotrexate

**DOI:** 10.3906/sag-1909-52

**Published:** 2020-08-26

**Authors:** Kübra KAVRAM SARIHAN, Melda YARDIMOĞLU YILMAZ, Fatma Ceyla ERALDEMİR, Yusufhan YAZIR, Esra ACAR

**Affiliations:** 1 Department of Histology and Embryology, Faculty of Medicine, Kocaeli University, Kocaeli Turkey; 2 Department of Medical Biochemistry, Faculty of Medicine, Kocaeli University, Kocaeli Turkey; 3 Stem Cell and Gene Therapies Research and Application Center, Kocaeli University, Kocaeli Turkey

**Keywords:** Testis, methotrexate, apocynin, apoptosis

## Abstract

**Background/aim:**

Methotrexate (MTX), widely used as a drug in cancer, has many adverse effects on tissues. Apocynin (APO) is a NADPH oxidase inhibitor and is known with many antioxidant properties. In this study, we aimed to evaluate the adverse effects of MTX on testicular tissue and the protective effects of APO at two different doses (20 mg/kg and 50 mg/kg) on MTX-induced testicular damage.

**Materials and methods:**

Fifty adult male Wistar albino rats (8 weeks old and weighing 200–250 g) were divided into five groups of 10 rats each: 1. saline control, 2. dimethyl sulfoxide (DMSO) control, 3. MTX, 4. APO-20 + MTX, and 5. APO-50 + MTX. All injections were performed intraperitoneally. At the end of day 28, all rats were sacrificed under anesthesia. The testes were evaluated histologically and the blood samples were analyzed biochemically.

**Results:**

According to histological and biochemical analyses, there was no significant difference between the DMSO and control groups. In terms of the histological findings, MTX group was significantly the worst affected group compared to the others, and in this group, apoptotic cell number (P = 0.011) was significantly increased in comparison with the control group. Except MTX, there was no significant difference in apoptotic cell number of the other groups compared to the control group. In the MTX group, malondialdehyde (MDA, P = 0.017) and myeloperoxidase (MPO, P < 0.001) levels were significantly increased in tissue and in blood (MDA P < 0.001, MPO P < 0.001), while tissue glutathione (GSH, P < 0.05) and serum testosterone levels (P < 0.01) were decreased compared with the control group. APO + MTX treatment groups exhibited better testis morphology, and apoptotic cells were also significantly decreased compared to MTX group (P < 0.001).

**Conclusion:**

Our results suggest that MTX induced defects on testis via oxidative stress and APO reversed the effects of MTX with its antioxidant properties.

## 1. Introduction 

One of the major causes of death in the world is cancer and in addition to surgery, immunotherapy, and radiotherapy, chemotherapy is one of the most widely used methods for the treatment.  However, chemotherapy treatments cause many undesirable side effects in multiple organ systems due to deleterious cytotoxicity against normal cells and tissues in the chemotherapy process [1]. Chemotherapeutic drugs often target rapidly growing cells. Therefore, the reproductive system is one of the most affected organs by chemotherapy. The chemotherapy applications induce oxidative toxicity and may lead to alteration in fertility, changing in organ structure, sexual hormones, and function [2,3].

 Methotrexate (N - 10 methyl aminopterin) is a folic acid antagonist having antiproliferative, antiinflammatory, and immunomodulatory effects and one of the main antimetabolites widely used in the treatment of many diseases including cancer, rheumatoid arthritis, and psoriasis [4–6]. Due to methotrexate’s (MTX) inhibitory effect on DNA synthesis, repair, and cellular replication, it also affects healthy tissues and cells, resulting in functional disorders of both somatic and reproductive cells [7,8]. Studies show that the MTX is cytotoxic to testicular and spermatogenic cells [9,10]. Several previous studies have reported that the MTX cause damage in the seminiferous tubules of testis, decrease in sperm number [11], and sperm DNA damage [12]. MTX induces the production of reactive oxygen radicals in cell and increased oxidative stress leads to damage to the structure of the testes and germ cells [13]. 

Apocynin (APO), obtained from *Apocynum cannabinum* extract, was first defined by Schmiedeberg in 1883 and was used for treatment of cardiovascular diseases as an antiedematous. APO is a selective members of the NADPH oxidase family inhibitor. It strongly inhibits NADPH oxidases by preventing the assembly of multisubunits [14]. NADPH oxidase enzyme is responsible for ROS production by catalyzing one electron reduction of molecular oxygen to generate O2–[u2926], which is a central and initial ROS molecule. Inhibition of this enzyme can be used as a treatment of numerous diseases like ischemia-reperfusion injury [15]. Therefore, APO is a very important antioxidant member to reducing oxidative stress of applications like chemotherapy. There is no study on the protective effect of APO on oxidative stress caused by MTX induction of the testis in the literature.

The aim of this study is to evaluate the protective properties of APO on testicular tissue as an antioxidant against the adverse effect of MTX which is widely used in the treatment of many diseases such as cancer.

## 2. Materials and methods

### 2.1. Animals

In this study, fifty adult Wistar albino rats (8 weeks old and weighing 200–250 g) were used according to the protocol approved by Kocaeli University Animal Experiments Local Ethics Committee (7 / 7- 2015). Animals were fed with ad libitum rat fodder and tap water in temperature (21 ± 2 °C)- and humidity-controlled rooms and exposed to a photoperiod of each 12 h day and night.

### 2.2. Experimental design

The animals were randomly divided into five groups, with 10 rats in each:

1) Saline control group: 0.9% NaCl (1.25 mL) was given to the rats in the control group [16].

2) Dimethyl sulfoxide (DMSO) control group: 15% DMSO (Santa Cruz Biotechnology, sc-202581-0.2 mL) was given to the rats in the DMSO group [17].

3) MTX group: The MTX group was administered a single dose of 20 mg/kg MTX (injectable flacon; 50 mg/5 mL; KoçakPharma Drug and Chemistry) as i.p. on the 24th day after starting the experiment [18]. APO was given to animals both before and after MTX administration. APO application was started on the first day of the trial period and continued until the 28th day. 

4) APO-20+ MTX group: 20 mg/kg APO (Santa Cruz Biotechnology, sc-203321A) was injected i.p daily for 4 weeks, after the experiment started [19] and a single dose of 20 mg/kg MTX was given i.p. on the 24th day after starting the experiment [18].

5) APO-50 + MTX group: 50 mg/kg APO was given i.p. daily for 4 weeks, after the experiment started [20] and a single dose of 20 mg/kg MTX was given i.p. on the 24th day after starting the experiment [18].

All rats were anesthetized with ketamine (90 mg/kg) + xylazine (10 mg/kg) and sacrificed 4 weeks after starting the experiment. Testes of all animals were removed, separated from adjacent tissues and then weighed. Right testes of animals were placed in 10% formaldehyde fixation solution for light microscopic examination. Left testes stored at –40 °C until assayed for malondialdehyde (MDA), myeloperoxidase (MPO), glutathione (GSH). In addition, blood samples were collected for serum testosterone determination.

### 2.3. Tissue processing for biochemical study

The tissues were weighed and homogenized with a tissue homogenizer by adding 1/10 (weight/volume) phosphate-buffered saline (PBS) (0.1 M/pH 7.4). Homogenates were centrifuged at 3000× *g* for 15 min and the supernatants were separated and stored in Eppendorf tubes at –40 °C until analysis. MDA and GSH levels were measured spectrophotometrically using the 1240 UV mini spectrophotometer (SHIMADZU) instrument with the manual method [19,21]. MPO levels were analyzed with Bioassay (Shanghai, China) ELISA kit using Alisei Elisa Reader (Italy, Rome). Tissue protein assay was performed with the Lowry method with modification [22]. MDA, GSH, MPO results were given as a ratio to tissue protein.

### 2.4. Blood samples for biochemical study

The blood samples were collected immediately from the heart and centrifuged at 3000× g for 15 min. The serum was stored at –40 °C for analysis of biochemical parameters. Serum MDA levels were determined by the method described by Buege and Aust [19]. Briefly, MDA was reacted with thiobarbituric acid by incubating for 15 min at 100 °C. MDA levels were measured spectrophotometrically at 535 nm using the 1240 UV mini spectrophotometer (SHIMADZU). Serum MPO levels were determined with sandwich ELISA kits on the basis of the manufacturer’s instructions (Bioassay Technology Laboratory, Shanghai, China) using Alisei Elisa Reader (Italy/Rome). Serum testosterone levels were measured in an autoanalyzer (Beckman Coulter, CA USA), an automated chemiluminescence immunoassay system. 

### 2.5. Tissue processing for histological study

For light microscopic examination, right testes were fixed in 10% buffered formalin for 48 h [23], and then processed, and then embedded in paraffin blocks. Ten serial sections were taken at 4 μm thickness from each paraffin block. Successive sections were stained with Hematoxylin-Eosin (H & E) [23] and terminal deoxynucleotidyltransferase-mediated dUTP nick end labeling (TUNEL) technique, and then examined, and photos were taken by a light microscope (Leica DM 1000, Leica DMC camera 2900). The testicular damage and apoptotic cells were assessed using H&E, and TUNEL assay technique for apoptosis. Histological damages were evaluated in sections stained with H&E according to previously reported procedures. Thirty seminiferous tubules were randomly examined in six fields of five sections for each rat. Four histological damages were scored in each seminiferous tubule as vacuolization, congestion, basal lamina undulation, and spilled germ cells in each seminiferous tubules. Seminiferous tubules showing no damage, mild damage, moderate damage, and extensive damage were scored as 0, 1, 2, and 3, respectively. The number of seminiferous tubules under each score was multiplied with the respective scores and the sum was obtained to get the final seminiferous tubule damage score [24].

### 2.5. Assessment of apoptotic cells with TUNEL (terminal deoxynucleotidyltransferase-mediated dUTP nick end labeling) assay for histological study

Paraffin sections were deparaffinized in toluene for 2 × 20 min, washed in PBS for 5 min after passing decreased alcohol series. Around the sections were drawn with a pap pen. The sections were processed for the TUNEL assay (An ApopTag Plus Peroxidase In situ Apoptosis Detection Kit, S7101; Merck Millipore Corp, Temecula, CA) according to the manufacturer’s instructions. The sections were incubated with a proteinase K solution for 15 min at room temperature for antigen retrieval and washed with distilled water (2 × 2 min). They were incubated with 3% H2O2 in methanol for 5 min to block endogenous peroxidase. After washing with PBS, the sections were incubated with equilibration buffer for 10 min and in terminal deoxynucleotidyl transferase enzyme under a humidified chamber at 37 °C for 60 min. The sections were incubated in stop buffer for 10 min and then washed in PBS (3 × 1 min), later incubated with antidigoxigenin-peroxidase conjugate for 30 min at room temperature and washed in PBS (4 × 2 min). They were incubated with DAB chromogen for 3–6 min, then rinsed with distilled water (3 × 1 min) and contrasted with 0.5% methylene green for 10 min. Later they were washed with distilled water (3 × 30 s) and submerged in N-butanol three times. The sections were cleared with toluene and mounted with Entellan (Merck, Germany). Twenty seminiferous tubules were randomly examined in each rat (5 section × 4 field). Brown-stained apoptotic germ cells were counted at randomly selected areas in each section under a light microscope (Leica DM 1000). The apoptotic index was calculated by counting the percentage of positive cells at 400× magnification [11,24].

### 2.6. Statistical analysis 

Statistical analysis was performed using SPSS 20.0 statistical software (IBM Inc, Chicago, IL, USA). Values were expressed as mean ± standard deviation (SD). The Kolmogorov–Smirnov test was used for evaluating the normal distribution of the variables. The difference between the groups was analyzed by one-way analysis of variance (ANOVA) for numerical variables with normal distribution, and with the Kruskal–Wallis test for numerical variables without normal distribution. In pairwise comparisons, the Mann–Whitney U-test was used after the Kruskal–Wallis test, and the Tukey test after the one way ANOVA test. Bonferroni correction was made in nonparametric tests. Tests were performed within 95% confidence interval and P-values of less than 0.05% were considered significant. 

## 3. Results 

### 3.1. Body and testis weights

There was not a significant difference between all groups in terms of body weight before MTX implementation (P = 0.226). At the end of the experiment, body weight of the animals was significantly increased in the control and APO + MTX treatment groups (P < 0.001). There was a significant decrease in body weights of MTX group (259.60 ± 16.60) compared to control (304.50 ± 8.54) and APO treatment groups, respectively (292.33 ± 22.18; 302.40 ± 9.54) (Table 1; P < 0.001).

**Table 1:** At the end of the experiment, the body weights and testicular weights of the rats for all groups are shown. Weights are expressed as mean ± standard deviation (SD).

Parametres	Saline control	DMSO control	MTX	APO-20+MTX	APO-50+MTX
Body weights (g)	304.50 ± 8.54	310.90 ± 12.05	259.60 ± 16.60**	292.33 ± 22.18++	302.40 ± 9.54++
Right testis (g)	1.78 ± 0.09	1.56 ± 0.12**	1.41 ± 0.11**	1.68 ± 0.07++	1.68 ± 0.07++
Left testis (g)	1.61 ± 0.11	1.55 ±0.15	1.33 ± 0.08**	1.69 ± 0.08++	1.69 ± 0.07++

*, ** Compare with saline control group; P < 0.05 and P < 0.001, respectively. +, ++ Compare with MTX group; P < 0.05 and P < 0.001, respectively

It was observed that right and left testicular weights were lowest in the MTX group (1.41 ± 0.11; 1.33 ± 0.08) and highest in control group, respectively (1.78 ± 0.09; 1.61 ± 0.11). There was no statistically significant difference between the control, APO-20+MTX (1.68 ± 0.07; 1.69 ± 0.08), APO-50+MTX (1.68 ± 0.07; 1.69 ± 0.07) treatment groups, but there was a significant difference between the MTX group and the other groups (Table 1; P < 0.001).

### 3.2. Biochemical examinations

#### 3.2.1. MDA and MPO levels

The MDA parameter was observed at higher levels in the MTX group as biochemical marker in tissue and serum, respectively (Tables 2 and 3; P < 0.05, P < 0.001). Tissue MDA levels were significantly different between control (5.01 ± 0.84) and MTX (8.59 ± 1.29), MTX and APO-20 + MTX (2.90 ±  0.86), MTX and APO-50 + MTX (5.58 ± 1.64) groups (P < 0.05; P < 0.001; Table 2). Serum MDA levels were also significantly different between control (1.71 ± 0.32) and MTX (6.54 ± 0.73), MTX and APO-20 + MTX (2.16 ± 0.53), MTX and APO-50 + MTX (1.72 ± 0.32) groups (P < 0.001; Table 3). 

**Table 2:** At the end of experiment, levels of MDA, MPO, and GSH in testicular tissues for all rat groups are shown. Data are expressed as mean ± standard deviation (SD).

Parametres	Saline control	DMSO control	MTX	APO-20+MTX	APO-50+MTX
MDA (nmol/mg protein)	5.01 ± 0.84	5.28 ± 1.05	8.59 ± 1.29*	2.90 ± 0.86**	5.58 ± 1.64+
MPO (ng/mg protein)	1.33 ± 0.43	1.57 ± 0.44	4.26 ± 0.79++	1.18 ± 0.67++	1.47 ± 0.34 +
GSH (mg/g protein)	14.04 ± 0.84	15.00 ± 2.53	10.58 ± 0.89**	16.74 ± 1.50**	17.07 ± 2.01**

*, ** Compare with Saline Control group; P < 0.05 and P < 0.001, respectively. +, ++ Compare with MTX group; P < 0.05 and P < 0.001, respectively

**Table 3:** At the end of experiment, levels of MDA, MPO, and testosterone in serum of all rat groups are shown. Data are expressed as mean ± standard deviation (SD).

Parametres	Salinecontrol	DMSO control	MTX	APO-20+MTX	APO-50+MTX
MDA (nmol/mL)	1.71 ± 0.32	2.09 ± 0.29++	6.54 ± 0.73**	2.16 ± 0.53++	1.72 ± 0.32++
MPO (ng/mL)	6.27 ± 1.30	7.56 ± 2.15	8.95 ± 1.45**	4.70 ± 0.52++	6.12 ± 0.56+
Testosterone (mg/mL)	5.05 ± 1.39	3.81 ± 0.77	1.49 ± 0.69**	6.27 ± 2.18++	5.61 ± 1.05++

*, ** Compare with saline control group; P < 0.05 and P < 0.001, respectively. +, ++ Compare with MTX group; P < 0.05 and P < 0.001, respectively

In MTX group, tissue (4.26 ± 0.79) and serum (8.95 ± 1.45) levels of MPO were higher than the levels of control (1.33 ± 0.43; 6.27 ±1.30), APO-20 MTX (1.18 ± 0.67; 4.70 ± 0.52), APO-50 MTX (1.47 ± 0.34; 6.12 ± 0.56), respectively. When the tissue and serum MPO levels were compared, there was no significant difference between control, DMSO, and APO-20+ MTX, APO-50 MTX groups (P < 0.05 and P < 0.001; Tables 2 and 3).

#### 3.2.2. GSH levels

The highest level of GSH was observed in APO treatment groups, on the contrary the lowest GSH level was seen in MTX group. There was a significant difference between control (14.04 ± 1.84) and MTX group (10.58 ± 0.89) in pairwise comparison (P < 0.001). GSH levels were significantly increased in the APO-20 + MTX (16.74 ± 1.50) and APO-50 + MTX (17.07 ± 2.01) groups when compared with the MTX group (Table 2; P < 0.001). 

#### 3.2.3. Serum testosterone levels

Serum testosterone levels were determined in blood samples obtained from cardiac puncture. The highest level of GSH was observed in both APO-20 + MTX (6.27 ± 2.18) and APO-50 + MTX (5.61 ± 1.05) groups while the lowest GSH level was seen in MTX (1.49 ± 0.69) group (Table 3; P < 0.001). There was no significant difference between the DMSO (P = 0.165), APO-20+MTX (P = 0.183) and APO-50+ MTX (P = 0.792) groups and the control group.

### 3.3. Histological examinations

**Figure 1:**
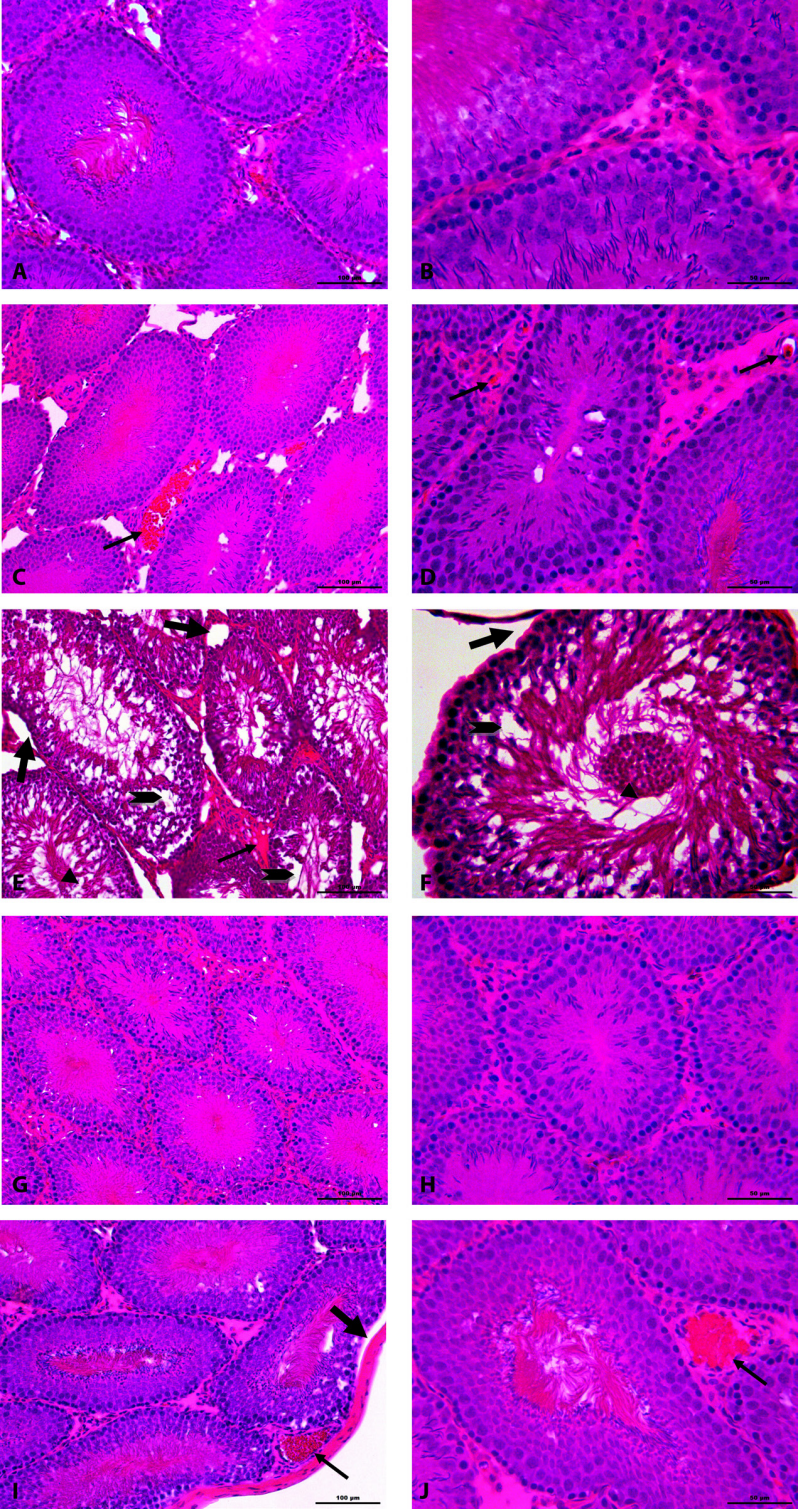
Photomicrograph of seminiferous tubules of (A, B) saline control, (C, D) DMSO control, (E, F) MTX, (G, H) APO-20 + MTX, (I, J) APO-50 + MTX groups. Note intact seminiferous tubules in Saline Control and DMSO Control groups with normal spermatogenesis. Congestion (thin arrow) in DMSO Control, MTX, APO-50 + MTX groups; seminiferous tubule with germinal epithelium dissociation and desquamated cells (arrow head), vacuolization ( ), basal lamina undulation (thick arrow) in the MTX group, and in APO-50 + MTX group continued vacuolization ( ). H&E staining, 200× (left column) and 400× (right column).

**Figure 2:**
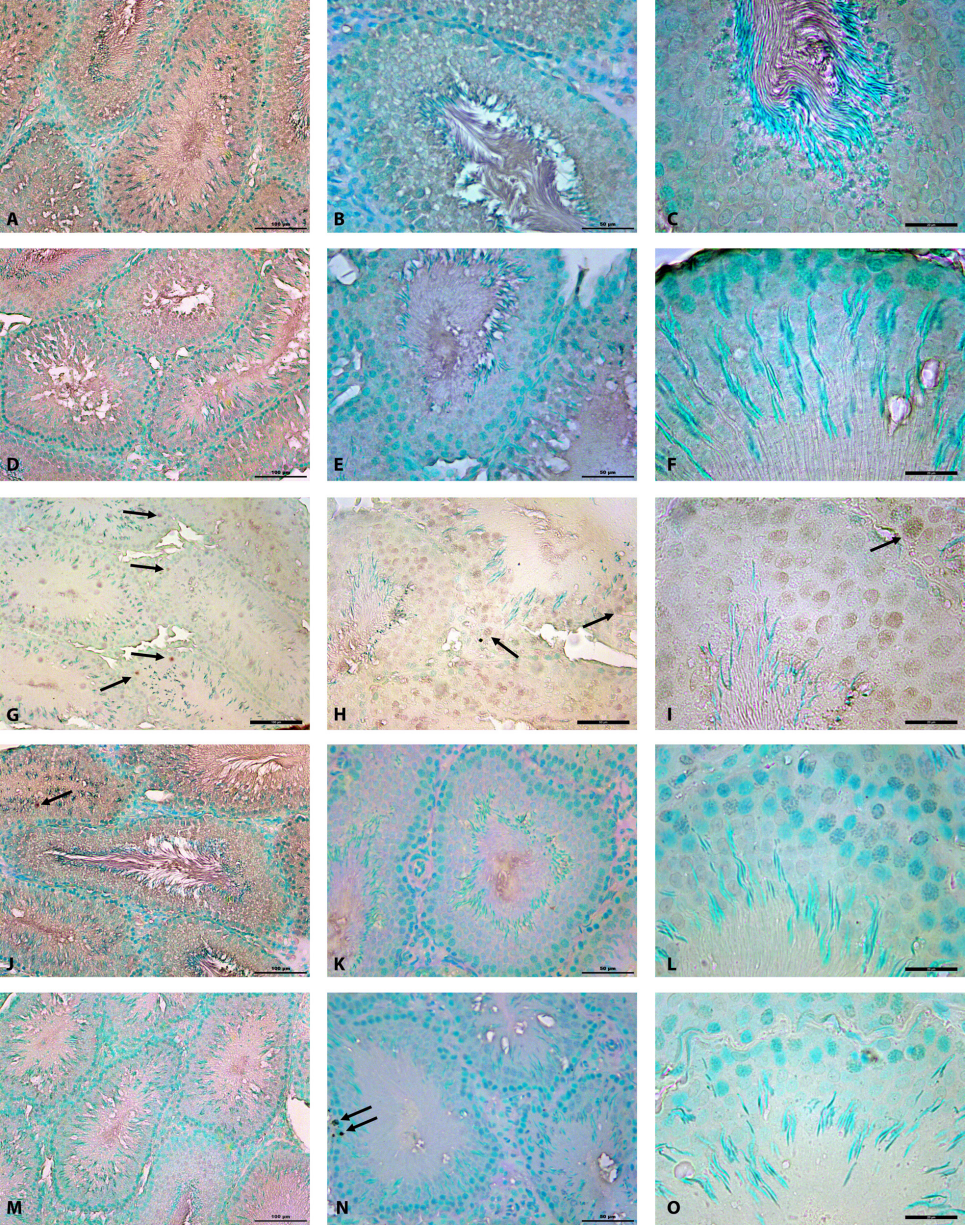
Photomicrographs of seminiferous tubules stained with TUNEL. Saline control group (A, B, C); DMSO control group (D, E, F); MTX group (G, H, I); APO-20 + MTX group (J, K, L); APO-50 + MTX group (M, N, O). Note increased apoptotic cells (arrow) in the MTX group (G-I), and decreased in APO treatment groups (J-O). 200×, 400×, and 1000× (left, middle, and right column, respectively).

**Figure 3:**
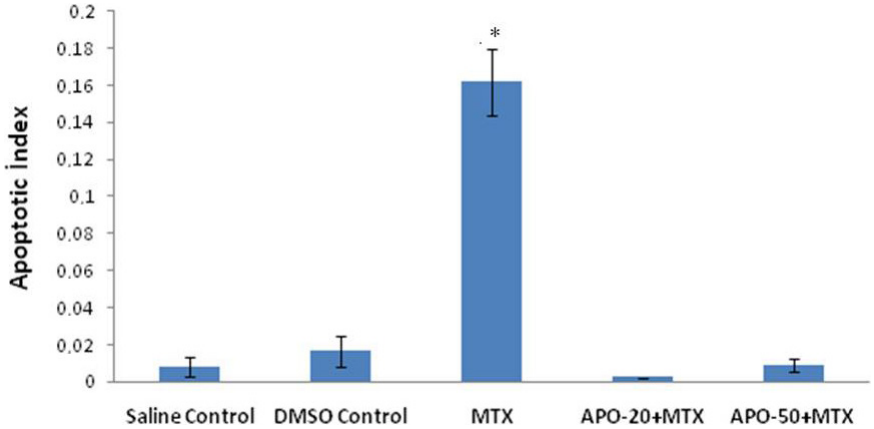
Column graph shows the comparison of apoptotic index (AI) according to experiment groups. * P <0.005; Compared to saline control group and MTX group; * P < 0.005; Compared to MTX and APO treatment groups.

Testis morphologies of control and DMSO groups were seen as normal including interstitial regions and seminiferous tubules (Figures 1A–1D). Vacuolizations in germinal epithelium, undulations in basal lamina, spilled immature germ cells in lumen of seminiferous tubules, and congestions in interstitial space were observed in MTX group (Figures 1E–1F). When the testicular damage was analyzed statistically in sections stained with H&E according to histological scores, there was a significant difference in terms of vacuolization, congestion, basal lamina undulation and spilled germ cells when MTX group compared with the control and DMSO groups (P < 0.001). In MTX group, the number of seminiferous tubules with histological damage increased significantly compared to the control group (P < 0.001). A regular interstitial area and seminiferous tubule histology were not observed in the MTX group (Figures 1E–1F). APO + MTX treatment groups exhibited a better morphological structure against MTX induced in germinal cell damage (Figures 1E–1J). Although both dose groups of APO continued to show a slight damage in a few seminiferous tubules, it was determined that tubular damage such as depletion and degeneration in germ cells, and congestion in interstitial region were not as intense as in the MTX group (Figures 1A–1J). Both APO-20 + MTX and APO-50 + MTX groups did not reveal a significant difference when compared to the control group for the vacuolization, basal lamina undulation, spilled germ cells, congestion (P = 1.000). A significant difference was determined when APO-20 + MTX group and APO-50 + MTX group compared to MTX group for the vacuolization criterion, basal membrane undulation, spilled germ cells and congestion (P < 0.001). When APO-20 + MTX group and APO-50 + MTX group were compared with each other, there was no difference (P = 1.000). A statistically significant difference was found between the groups according to TUNEL technique (P = 0.002). There was a significant difference between the control group and the MTX group (P = 0.011). In MTX group, apoptotic cells were seen more compared to the other groups (Figures 2 and 3). There was also a significant difference between MTX group and both dose groups of APO. AI was significantly decreased in APO + MTX treatment groups compared with the MTX group (Figure 3).

## 4. Discussion

The present study represented for the first time that APO has a protective effect on MTX-induced testicular damage. According to earlier studies, chemotherapeutic drugs have been reported to have many side effects [11,16,24–27]. Oxidative stress is one of the major factors in MTX-induced damage for many organs [28–31]. The most important mechanism in the formation of tissue damage due to free oxygen radicals is the peroxidation of lipids (LPO) in cell membranes [32] and LPO can be used as a marker of tissue damage. MDA is one of the degradation products of LPO. Therefore, MDA levels are also an important predictor of free radical production [33]. The marked increase of MPO and MDA in serum and testicular tissue in MTX group observed in this study complies with previous studies attributed to oxidative stress in MTX‐mediated toxic injury [18,31,34–37]. Adding an antioxidant medication to MTX treatment regimen may be the solution to reduce or prevent the side effects of MTX. Many researchers have studied effect of different substances such as against oxidative damage of MTX in testes [10,11,18,31,34,37–44]. MTX + caffeic acid phenethyl ester (CAPE) treatment caused the oxidative stress to increase in the testicles despite the antioxidant, antiinflammatory properties of CAPE [31]. In a study by Vardı et al. [39], the antioxidant and antiapoptotic effects of β-carotene against MTX-induced testicular injury, MDA levels in the MTX group have been seen significantly higher than the β-carotene + MTX group. In another antioxidant experimental study, MDA levels in the MTX group were found to be significantly higher than the Resveratrol + MTX group [11]. Güvenç and Aksakal [36] concluded that there was an increase in MDA levels and a decrease in GSH levels in the MTX group, and MTX-induced reproductive toxicity was reduced by an antioxidant compound, Kisspeptin. The results obtained by the researchers described above regarding MTX are similar to our findings. MTX reduces the effectiveness of the antioxidant enzyme system causing the cells to become susceptible to ROS and eventual cellular injuries. In our study, there was a significant difference between the control and MTX group and treatment groups and MTX group in terms of MDA levels. There was an increase in MDA levels in tissue and serum due to increase of oxidative stress in MTX group while a decrease in APO + MTX groups, especially an important decrease in APO-20 + MTX group in tissue MDA with the improvement in histological appearance. Our findings showed that MPO and MDA levels in MTX + APO treatment groups decreased significantly with the antioxidant properties of APO, similar to previous studies [17,45].

Many studies have reported a decrease in testicular tissue and serum levels of GSH, which has a protective role on tissues in rats treated with MTX [10,17,31,34,36–40,44,46]. GSH levels decreased in also I/R-damaged rats and increased in the APO treatment groups. It was shown that the increase in superoxide radicals results in excessive consumption and reduction of antioxidant-affecting thiols such as GSH [45]. Şener et al. [17] determined that MDA levels were decreased in APO treatments at doses of 20 mg/kg and 50 mg/kg against I/R; APO treatment has been reported to be effective in both doses but 50 mg/kg more effective for an increase in GSH. In our study, MTX directly suppressed the GSH defense system. GSH levels in both dose of APO treatment groups were markedly increased with antioxidant properties of APO similar to previous studies [ 35,37,45–50]. 

In our study, the observed decrease in body and testis weight in MTX-treated group due to the oxidative stress occurred was in accordance with previous studies [24,31,36,40,41]. We found that rats treated with MTX exhibited a reduction in testicular weight parallel with a decrease in serum testosterone levels and increase in testis damage. There was a relationship between decreased testicular weight and histopathological damage including increased apoptotic index. Although Leydig and Sertoli cells are known as more resistant than germinal cells, these drugs negatively affect them too; such as disrupted of protein synthesis, microfilament, microtubule, and cell adhesion in Sertoli cells. Therefore, MTX can also damage the intercellular junctional complexes and cause the cells to shed into the lumen. The negative deterioration in this cycle is reflected in total as a decrease in testicular weights [35,36,40]. It has been reported that damage caused by MTX to spermatogenic cells could lead to infertility [9,25,36]. The main reason of decreased testosterone level is oxidative stress-induced testicular damage by MTX on mainly Leydig cells, and spermatogenic cells, Sertoli cells, and results in impaired function and a decrease in testosterone level [25,27,37,40,51–54]. In previous studies, testosterone levels in chemotherapy groups (cisplatin, MTX) group were significantly decreased, however, in treatment groups (cisplatin + curcumin, cisplatin + GH) were increased significantly [41,51]. APO due to similar effects of curcumin, which has an antioxidant and antiinflammatory effect, our findings regarding testosterone are similar to those of these studies. The negative effects of MTX on testosterone appear to be eliminated in both doses of APO. APO prevents the formation of free oxygen radicals and apoptosis by inhibiting NADPH oxidase [14,45,46,53].

Li et al. [20] reported that testosterone levels decreased significantly because of the oxidative stress on the testis of diabetic rats, on the contrary in the APO (16 mg/kg per day) groups increased significantly. They concluded that APO significantly reduced the production of ROS and apoptotic cells and increased the total testosterone level. In our study, serum testosterone levels were significantly lower in the MTX group compared with the control and APO treatments groups. In both doses of APO treatment, serum testosterone levels were significantly higher. Our findings agree with those of previous studies [38–40]. This study showed that there were no significant differences with respect to testicular and body weight, and testosterone levels between the control and APO treatment groups. Our results demonstrated that the administration of APO reduced the effect of MTX-induced decline in testosterone hormone levels and decreased oxidative stress markers markedly. Thus, APO improves testis structure with endocrine balance to reduce MTX‐induced testicular toxicity. Liu et al. [46] reported that APO increased the antioxidant defense system, and GSH also reduced cellular stress. Çağın et al. [49] treated rats at a dose of 20 mg/kg for 3 consecutive days by APO against cisplatin toxicity. They found that the LPO and total oxidant status were significantly increased and SOD and glutathione peroxidase levels were significantly decreased when compared with controls. In another study by the same authors treated rats with a single dose of 20 mg/kg APO for 5 days against ionizing radiation. They reported that APO prevented oxidative stress and apoptotic cell death by inhibiting NADPH oxidase, and preventing the formation of ROS. Moreover, their results are consistent with ours that APO might have a protective effect [50]. Low-dose (200 μg/mL) and high-dose (1000 μg/mL) of APO have been given to mice for 30 days in an oligoasthenozoospermia model. The authors stated that APO could significantly improve sperm quality in these cases by eliminating ROS and improving antioxidant activity of the body; hence, it can be applied in the prevention and treatment of male infertility [54]. 

Apoptosis is known to play an important role on spermatogenesis in human testis [55–57], and the increase of apoptosis cause deterioration of spermatogenesis [30]. Numerous studies have reported that apoptosis is an important indicator of damage to spermatogenic cells by drugs of chemotherapy [11,32,35,58]. Chemotherapeutic drugs such as cisplatin, Adriamycin and MTX caused the depletion and degeneration of germ cells in rats [10,11,13,24,31,34,35,58,59]. It is recommended that treatment with MTX may be combined with some medicines to prevent side effects on these cells. In this regard, there are many studies in the literature on antioxidant substances such as vitamin E and resveratrol used against MTX toxicity. Therefore, compounds with antioxidant properties may protect testicular tissue from harmful effects of the oxidative stress caused by MTX [11,31,36,39].

In our study, we determined that apoptotic cells are induced by MTX, and apoptotic index decreases with APO treatment. Like cisplatin and adriamycin, MTX showed effects such as causing germ cells damage, vacuolization, and spillage in the seminiferous epithelium. Spermatogenic cells are quite susceptible to detrimental effects of chemotherapeutics, and the most susceptible cells are dividing spermatogonia and spermatocytes. Therefore, in our study, apoptotic cells were most frequently observed at this stage. In a study on mice, it was shown that apoptotic cells were increased in the MTX-treated group, while dose-dependent folic acid was given before MTX and apoptotic cells were decreased in dose-dependent manner in folic acid groups after MTX treatment. Thus, the toxic effect of MTX on the germ cells has been demonstrated to be related to folic acid depletion [10,24,31,37–40,43,44]. In another study, it was shown that apoptotic cells decreased in the group receiving thymoquinone + MTX [43]. Likewise, it was reported that apoptotic cells were reduced in rats after resveratrol treatment [11]. It has been recommended that cisplatin-induced testicular apoptosis could be prevented by administration of curcumin, vitamin E, and combination therapy of antioxidants [58]. MTX-induced germ cell toxicity has been studied at 5 mg/kg once a week for 4 consecutive weeks. Several histopathological alterations in the testis such as desquamation, altered tubular structures, and DNA damage (detected with TUNEL assay) have been reported [44]. In another study, it has been recommended that cisplatin-induced testicular apoptosis could be prevented by administration of curcumin, vitamin E, and combination therapy of antioxidants [58]. Belhan et al. [10] reported that the effects of MTX such as degenerative changes in the testicles were decreased with the administration of hesperidin, which has antioxidant and antiinflammatory effects. Examination of the testes demonstrates that the reduction of antioxidant status resulted in apparent oxidative and testicular damages in MTX‐treated animals. As seen from our histological examinations in testes, MTX caused severe damage such as the formation of vacuolizations in germinal epithelium, undulations in basal lamina, spilled immature germ cells in lumen of seminiferous tubules, congestions in interstitial space, and increased apoptotic index, and APO significantly improved these changes. Our findings support other studies on the effects of MTX [32]. Although apoptosis increased in the MTX group, it decreased in the MTX + APO treatment groups, suggesting an antiapoptotic feature of APO. It was determined that testicular damage increased parallel to decreasing testosterone and GSH levels. In our study, MTX caused structural damages in the testis by inducing oxidative stress and both doses of APO ameliorated tissue damage with the antioxidant effect. Therefore, when MTX is to be used as a drug for treatment, APO may be used as an antioxidant to prevent the damaging effect of MTX. Although the effects of these doses are similar according to biochemical parameters, we can say that the APO-20 mg/kg dose gives better results when the histological structure of the seminiferous tubules is examined. 

In conclusion, while the total body weights and testicular weights were reduced greatly in MTX-treated rats, these losses were restored in MTX + APO treatments. MDA and MPO levels and GSH consumption were significantly reduced by APO treatment indicating that treatment with APO significantly reduces MTX-induced testicular damage. Examination of testes with H&E staining and TUNEL technique in light microscopy indicates that administration of APO has a protective effect on MTX-induced testicular damage. The present study suggests that APO had a chemoprotective effect, possibly due to its antiapoptosis and antioxidant properties on the testicles. Further multidisciplinary and pharmacological studies are needed to use APO as a drug.

## Acknowledgments

The authors would like to thank Professor Dr. Ali SAZCI from Kocaeli University Department of Medical Biology for editing the English of our manuscript. This study was financially supported by the Scientific Research Foundation of Kocaeli University Scientific Research Foundation, Grant number: 2015/59HD, Kocaeli, Turkey.

## Abbreviations

MTX: Methotrexate; ROS: reactive oxygen species; APO: Apocynin; NADPH: Nikotinamid adenin dinükleotit fosfat; GSH: Glutathione; DMSO: Dimethyl sulphoxide; MDA: Malondialdehyde; MPO: Myeloperoxidase; LPO: Lipit Peroxidation; PBS: Phosphate buffered saline; H&E: Haematoxylin & Eosin; TUNEL: Terminal deoxynucleotidyl transferase-mediated dUTP nick end labeling; I/R: Ischemia-Reperfusion; NO: Nitric oxide; SOD: Superoxide dismutase.

## Authors’ contributions

1) Kübra Kavram Sarıhan was responsible for %42 of this project. Her contributions were to prepare solutions, treatments, collection of blood and tissues, take in fixatives, etc. and to perform immunohistochemical studies. 

2) Melda Yardimoglu Yılmaz was the mentor of thesis; She was responsible for %43 of this project. Her contibutions were to determine and prepare laboratory equipment and tasks like immunohistochemical studies, evaluation of results and discussion, work design, and manuscript writing.

3) Fatma Ceyla Eraldemir was responsible for %5 of this project. She helped in biochemical technical matters and evaluation. 

4) Yusufhan Yazır was responsible for %5 of this project (technical equipment) .

5) Esra Acar was responsible for %5 of this project. She helped in technical matters and in preparing biochemical procedures.

Concept: MY, KKS; Design: MY, KKS, CE; Supervision: MY, KKS, EA; Resource: MY, KKS; Materials: KKS, YY.

Data collection and/or processing: KS, CE, EA, MY; Analysis and/or Interpretition: MY, CE, EA; Literature review: MY, KKS; Writing: MY, KKS, CE, E. Critical reviews: MY, CE, EC.
